# Association between mean corpuscular volume and mortality in chronic kidney disease ICU patients: A retrospective multicenter cohort study

**DOI:** 10.1371/journal.pone.0328980

**Published:** 2025-08-13

**Authors:** Sheng Chen, Yiwen Li, Lin Guo, Xiaohan Ma, Xuejin Ye, Lingling Wu, Ting Zhang, Hongjun Gao

**Affiliations:** 1 Graduate School, Guangxi University of Chinese Medicine, Nanning, Guangxi, China; 2 Ruikang Hospital, Guangxi University of Chinese Medicine, Nanning, Guangxi, China; Ross University School of Veterinary Medicine, SAINT KITTS AND NEVIS

## Abstract

**Background:**

Chronic kidney disease (CKD) affects over 10% of the global population and is closely linked to increased cardiovascular morbidity and mortality. Mean corpuscular volume (MCV), a key hematological parameter, has been associated with various clinical outcomes. However, the relationship between MCV and mortality in CKD patients admitted to the intensive care unit (ICU) has not been thoroughly investigated, with previous studies primarily limited to single-center designs.

**Methods:**

This retrospective multicenter cohort study analyzed data from the eICU-CRD and MIMIC-IV databases. Statistical analyses involved Kaplan-Meier survival curves and multivariable Cox proportional hazards models. Restricted cubic splines (RCS) were employed to assess the potential nonlinear relationships between MCV and mortality.

**Results:**

A total of 23,724 patients were included in the analysis. Higher MCV levels were significantly associated with increased 30-day and 90-day in-hospital mortality. Kaplan-Meier analysis revealed a higher mortality risk in patients with the highest MCV levels. Cox models confirmed that MCV was a significant risk factor for mortality, with hazard ratios indicating an increased risk with each unit increase in MCV. Subgroup analyses consistently showed that elevated MCV levels were associated with a higher mortality risk across different patient groups.

**Conclusion:**

This first multicenter study demonstrated that elevated MCV levels are significantly associated with higher short-term mortality in CKD ICU patients, suggesting that MCV could serve as a potential biomarker for risk stratification. Future research should validate these findings and explore the underlying mechanisms.

## 1. Introduction

Chronic kidney disease (CKD) represents a significant and growing public health challenge that affects over 10% of the global population, making it one of the most prevalent and concerning health conditions worldwide. The disease is primarily characterized by a progressive and irreversible loss of nephron function, which over time leads to a decline in the kidney’s ability to filter blood effectively. This nephron loss is accompanied by a series of pathological changes in the kidney, including tubular atrophy, interstitial fibrosis, glomerulosclerosis, vascular rarefaction, and arteriosclerosis, all of which contribute to the gradual worsening of kidney function and ultimately lead to end-stage renal disease in many affected individuals [1,2].

MCV is an important hematological parameter that measures the average size of red blood cell (RBC) and is widely used in the diagnosis and classification of anemias [3]. Recently, associations between MCV and patient outcomes have been reported in individuals with various conditions, including HIV [4], gastroesophageal adenocarcinoma [3], colorectal cancer [5], liver cancer [6], and acute decompensated heart failure [7]. Additionally, MCV has been linked to the prognosis of patients with CKD. Higher MCV levels have been associated with increased all-cause, cardiovascular, and infection-related mortality in individuals with CKD [8].

Despite the growing recognition of the importance of various biomarkers in predicting clinical outcomes, there remains a notable lack of comprehensive research exploring the relationship between MCV levels and mortality risk in critically ill patients with CKD who are admitted to ICU. While some studies have investigated the link between MCV and mortality risk in patients with CKD, most of these studies have been conducted within single-center settings, which significantly limits the external validity and generalizability of their findings to broader, more diverse patient populations. This limitation is important, as the variability in patient demographics, clinical practices, and healthcare resources across different centers could potentially influence the results.

To address this critical gap in the literature, the present study was specifically designed to explore the association between MCV levels and mortality outcomes in ICU patients with CKD, using a multicenter approach that enables a more robust analysis and enhances the generalizability of the findings across a wider range of healthcare settings and patient populations.

## 2. Materials

### 2.1. Data source

This retrospective cohort study made use of data sourced from two prominent databases: the eICU Collaborative Research Database (eICU-CRD) and the Medical Information Mart for Intensive Care (MIMIC-IV). The eICU-CRD is a large-scale, multicenter telemedicine database that compiles comprehensive clinical data from over 200,000 patients who were admitted to a total of 335 ICUs across 208 hospitals located throughout the United States. This extensive dataset allows for a diverse representation of critically ill patients and enables researchers to conduct analyses across a broad range of clinical settings, which increases the robustness and applicability of the findings [9].

In addition, the MIMIC-IV database offers detailed information on all patients who received treatment at the Beth Israel Deaconess Medical Center. This database provides a wealth of data on a large cohort of patients, which helps to enhance the comprehensiveness of the study and enables an in-depth examination of various clinical outcomes, making it a valuable resource for research on critical care [10].

In accordance with the established protocols and ethical guidelines for the use of the MIMIC and eICU-CRD databases, one of the authors of this study, Sheng Chen, successfully completed a comprehensive human subject research training course, which was designed to ensure adherence to ethical standards when handling patient data. This course, identified by the record ID 66963781, provided the necessary educational foundation on human subject research ethics and best practices in data handling. Subsequently, Sheng Chen achieved the credentialed user status on PhysioNet, which is a necessary requirement for gaining authorized access to the databases.

The studies involving human participants were reviewed and approved by MIMIC-IV databases were approved by the institutional review boards of the Massachusetts Institute of Technology and Beth Israel Deaconess Medical Center. Written informed consent for participation was not required for this study in accordance with the national legislation and the institutional requirements.

Upon completion of the training and achieving the necessary credentials, our research team officially signed the Data Use Agreement, a formal contract that outlines the responsibilities and terms for accessing and utilizing the data in a secure and ethical manner. Following the signing of this agreement, our team was granted the appropriate authorization, allowing us to access both the MIMIC-IV and eICU-CRD databases. This process ensured that all data usage complied with institutional and legal guidelines and that the research was conducted with the utmost respect for patient privacy and confidentiality.

The database contains a wide range of detailed and comprehensive patient information, encompassing key clinical data such as the duration of hospital stays, results from various laboratory tests, records of medication administration, and monitoring of vital signs, which are essential for understanding the clinical course and treatment of critically ill patients. These data provide valuable insights into the management of patients in intensive care settings, allowing for an in-depth analysis of their conditions and outcomes. In order to ensure the protection of patient privacy and confidentiality, all personally identifiable information within the database was systematically de-identified.

This process involved replacing any direct identifiers with randomly generated codes, thus ensuring that individual patients could not be traced or identified through the data. As a result of these measures, the study did not require explicit patient consent or formal ethical approval, as the data utilized was anonymized, reducing the risk of violating patient privacy rights. These practices are in line with standard protocols for the use of large healthcare databases in research, ensuring both compliance with ethical standards and safeguarding patient confidentiality.

### 2.2. Study population

Patients who had been diagnosed with CKD were included in the analysis based on the diagnostic criteria established in the 9th and 10th revisions of the International Classification of Diseases (ICD-9 and ICD-10), which provide standardized codes for the identification of CKD and ensure consistency in diagnosing and classifying patients with this condition. However, several specific patient groups were excluded from the study to ensure that the sample population was appropriate for the research objectives and to reduce potential biases.

The exclusion criteria were as follows: (1) individuals who were under 18 years of age, as pediatric patients may have different disease progression and treatment outcomes compared to adults; (2) patients who had multiple ICU admissions during the study period, with only the first admission being considered for analysis in order to avoid bias introduced by repeated hospitalizations. (3) patients whose ICU stay was shorter than 3 hours, as such brief stays are unlikely to provide meaningful clinical data or reflect the typical course of treatment and outcomes in critically ill CKD patients; (4) patients who did not have a recorded MCV measurement, as this key laboratory value was necessary for the study’s analysis of its association with mortality risk; and (5) patients who had missing data for other relevant covariates, as incomplete information could skew the results and undermine the accuracy of the study’s findings. These exclusion criteria were designed to ensure that the study population consisted of patients who met the necessary conditions for robust and valid analysis.

### 2.3. Data extraction and processing

In this study, the maximum value of the patient’s MCV recorded within the previous 24 h of monitoring was extracted. Additional covariates included demographic information, such as age, sex, weight, and race, which provided a foundational profile of the patient cohort.

Comorbidities including heart failure, respiratory failure, atrial fibrillation, paraplegia, diabetes, sepsis, and stroke were also considered, all of which may influence patient outcomes. Laboratory parameters measured within the first 24 h of ICU admission were also included, such as RBC and white blood cell (WBC) counts, hemoglobin and platelet levels, serum sodium and creatinine levels, and fasting blood glucose (FBG) levels. These laboratory measures offer valuable insights into patients’ physiological and metabolic states, which are essential for assessing disease severity and prognosis.

Illness severity scores assessed on ICU admission were also considered, including the Simplified Acute Physiology Score II (SAPS II), Oxford Acute Severity of Illness Score (OASIS), Charlson Comorbidity Index (CCI), and Sequential Organ Failure Assessment (SOFA). These scoring systems are standard in clinical practice for evaluating disease severity and predicting patient outcomes, and thus provide crucial information for analysis [11,12].

Finally, medication use in the ICU, including epinephrine, dopamine, and vasopressin, was recorded.

### 2.4. Clinical outcomes

The primary endpoints of this study were the 30-day and 90-day mortality rates during hospitalization. The secondary endpoint was all-cause mortality during the hospital stay.

### 2.5. Statistical analysis

Continuous variables in the study were summarized and described using medians along with interquartile ranges, as these measures provide a robust representation of the central tendency and spread of data, especially in the presence of skewed distributions. For comparisons between the two groups, the Mann-Whitney U test was applied, as it is a non-parametric test that is well-suited for comparing differences between two independent groups when the assumptions of normality are not met.

Categorical variables, which represent distinct categories or groups, were presented as counts and percentages, providing a clear overview of the distribution of different groups within the sample. To evaluate differences between these categorical variables, either the chi-square test or Fisher’s exact test was applied, depending on the frequency distribution of the categories. These statistical tests are commonly used to determine whether there are significant associations or differences between groups with categorical data.

The Kaplan-Meier method was employed to construct survival curves, which were used to estimate and visualize the probability of survival over time, specifically focusing on 30-day and 90-day mortality outcomes. This method is widely recognized for its ability to handle censored data and provide a clear depiction of the survival experience of patients over a specified time frame.

To further explore the relationship between MCV and in-hospital mortality at both 30 days and 90 days, multivariable Cox proportional hazards models were utilized. These models allow for the examination of the impact of MCV, adjusting for potential confounders, and are well-suited for investigating the time-to-event data associated with mortality outcomes in critically ill patients with CKD.

Furthermore, to address the possibility of nonlinear relationships between MCV levels and the primary mortality outcomes, RCS were incorporated into the analysis. RCS is a flexible approach that enables the modeling of complex, nonlinear associations, ensuring a more accurate and nuanced understanding of how MCV may influence mortality over time.

To verify the robustness of our results. We performed multiple sensitivity analyses. All statistical analyses were conducted using R 4.2.3, with a p-value of <0.05 considered statistically significant.

## 3. Results

### 3.1. Study participants and baseline characteristics

**Fig 1:**
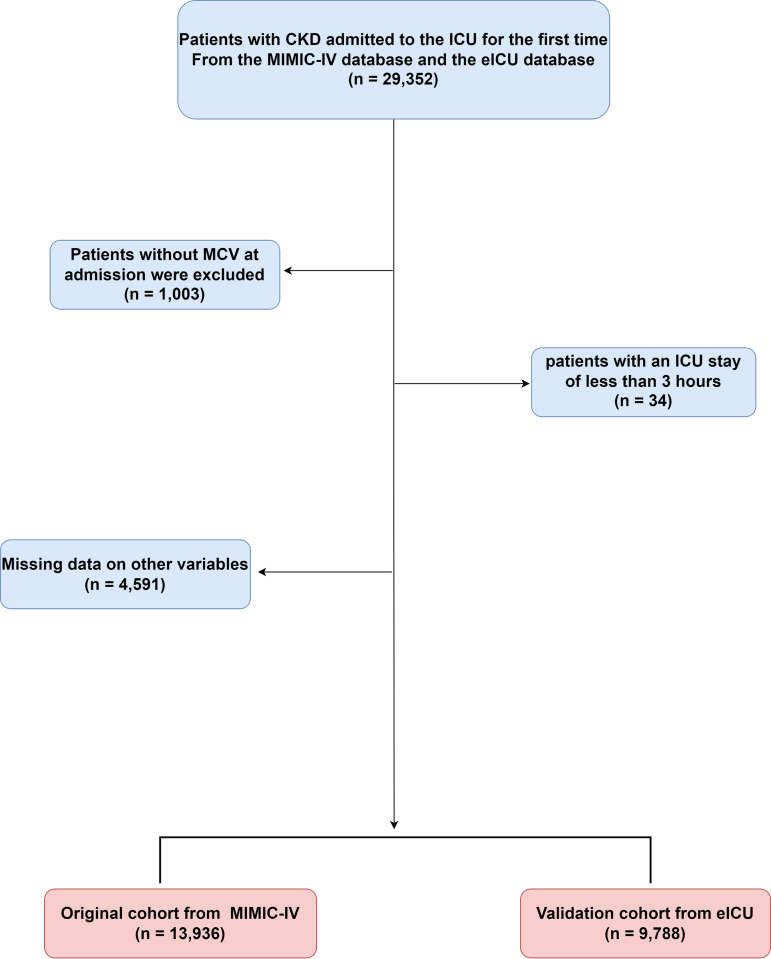
Flowchart of the study population.

After carefully applying the predetermined exclusion criteria to remove patients who did not meet the necessary requirements for the study, a total of 23,724 patients were ultimately included in the analysis. This cohort comprised 13,936 patients who were part of the original patient group extracted from the MIMIC-IV database, which provided a diverse set of clinical data from a range of healthcare settings. Additionally, the analysis also included 9,788 patients from the validation cohort, which was extracted from the eICU-CRD database. The inclusion of two separate cohorts, one from each of these large, multicenter databases, allowed for a more comprehensive examination of the research question, enhancing the generalizability and robustness of the study’s findings (Fig 1).

The baseline characteristics of all patients included in the analysis are presented in detail in Table 1 and S1 Table, providing an in-depth description of the demographic, clinical, and laboratory variables for the study population. These tables serve to give a clear overview of the distribution of key characteristics, allowing for comparison and understanding of how these variables might influence the study’s outcomes.

**Table 1:** Baseline characteristics of survivors and deceased patients in the original cohort.

Variable	Total (n = 13936)	Alive (n = 12080)	Death (n = 1856)	p.value
MCV	93.25 ± 7.63	92.95 ± 7.45	95.23 ± 8.45	<0.0001
Sex				0.69
Female	5364(38.49)	4658(38.56)	706(38.04)	
Male	8572(61.51)	7422(61.44)	1150(61.96)	
Age (years)	73.40(63.38,82.20)	72.80(62.75,81.77)	76.72(67.72,84.25)	<0.0001
Weight(kg)	80.00(67.80,96.00)	80.40(68.00,96.30)	77.75(66.00,93.85)	<0.0001
Comorbidities				
Heart failure				<0.0001
No	6209(44.55)	5519(45.69)	690(37.18)	
Yes	7727(55.45)	6561(54.31)	1166(62.82)	
Respiratory failure				<0.0001
No	9345(67.06)	8661(71.70)	684(36.85)	
Yes	4591(32.94)	3419(28.30)	1172(63.15)	
Arterial fibrillation				<0.0001
No	8052(57.78)	7145(59.15)	907(48.87)	
Yes	5884(42.22)	4935(40.85)	949(51.13)	
Diabetes				<0.01
No	6486(46.54)	5561(46.03)	925(49.84)	
Yes	7450(53.46)	6519(53.97)	931(50.16)	
Paraplegia				0.72
No	13879(99.59)	12032(99.60)	1847(99.52)	
Yes	57(0.41)	48(0.40)	9(0.48)	
Sepsis				<0.0001
No	10874(78.03)	9952(82.38)	922(49.68)	
Yes	3062(21.97)	2128(17.62)	934(50.32)	
Stroke				<0.0001
No	13401(96.16)	11661(96.53)	1740(93.75)	
Yes	535(3.84)	419(3.47)	116(6.25)	
TIA				1.00
No	13895(99.71)	12044(99.70)	1851(99.73)	
Yes	41(0.29)	36(0.30)	5(0.27)	
Laboratory tests				
Hemoglobin, g/dL	9.87 ± 1.81	9.88 ± 1.79	9.83 ± 1.95	0.31
Platelet, K/uL	179.00(132.00,240.00)	180.00(134.00,239.00)	172.00(117.00,245.00)	0.03
RBC, m/uL	3.34 ± 0.65	3.35 ± 0.64	3.32 ± 0.70	0.15
WBC, K/uL	10.40(7.50,14.60)	10.10(7.40,14.00)	13.00(8.80,18.20)	<0.0001
SCr, mg/dL	2.10(1.40,3.70)	2.00(1.40,3.60)	2.60(1.80,4.00)	<0.01
FBG, mg/dL	131.00(105.00,175.00)	130.00(104.00,172.00)	144.00(111.00,203.00)	<0.0001
Sodium, mEq/L	138.70 ± 5.00	138.66 ± 4.81	138.91 ± 6.11	0.09
SOFA	5.00(4.00,8.00)	5.00(3.00,7.00)	8.00(6.00,11.00)	<0.0001
SAPSII	40.00(33.00,49.00)	39.00(32.00,47.00)	51.00(42.00,63.00)	<0.0001
OASIS	32.29 ± 8.73	31.30 ± 8.20	38.72 ± 9.33	<0.0001
CCI	7.92 ± 2.39	7.79 ± 2.36	8.79 ± 2.37	<0.0001
Drug use				
Epinephrine				<0.0001
No	13133(94.24)	11519(95.36)	1614(86.96)	
Yes	803(5.76)	561(4.64)	242(13.04)	
Dopamine				<0.0001
No	13370(95.94)	11707(96.91)	1663(89.60)	
Yes	566(4.06)	373(3.09)	193(10.40)	
Vasopressin				<0.0001
No	12773(91.65)	11479(95.02)	1294(69.72)	
Yes	1163(8.35)	601(4.98)	562(30.28)	

Data are presented Standard Deviation (SE) or frequencies (percentages).

Abbreviation: MCV, mean corpuscular volume; SOFA, sequential organ failure assessment; CCI, Charlson comorbidity index; SAPSII, simplified acute physiological score II; OASIS, oxford acute severity of illness score; WBC, white blood cell; RBC, red blood cell; FBG, fasting blood glucose

Regarding the in-hospital mortality rates, the data revealed that the mortality rate for patients in the original cohort was 13.32%, reflecting the severity and complexity of the clinical conditions observed in this group. For the validation cohort, which was derived from a different database, the in-hospital mortality rate was slightly lower at 12.47%, though still indicative of the high-risk nature of the ICU patients with CKD. These mortality rates highlight the serious challenges in managing critically ill patients with CKD, underlining the importance of identifying risk factors and biomarkers, such as MCV, that may influence patient outcomes.

In the original cohort, deceased patients had higher values for weight, WBC, serum creatinine, blood glucose, SOFA, SAPS II, OASIS, and CCI than survivors, while hemoglobin, platelet, and RBC levels were lower in deceased patients. A similar pattern was observed in the validation cohort. Notably, the MCV of survivors was higher than that of deceased patients in both cohorts (p < 0.01).

### 3.2. Associations between MCV levels and patient outcomes

#### 3.2.1. Kaplan-Meier survival analysis curves for mortality.

**Fig 2:**
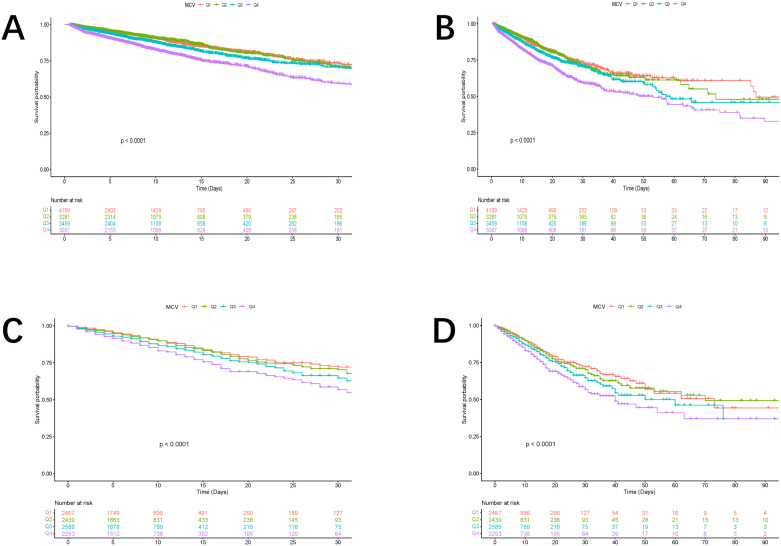
Kaplan-Meier curves showing the cumulative probability of mortality. (A) Death within 30 days in the original cohort; (B) death within 90 days in the original cohort; (C) death within 30 days in the validation cohort; and (D) death within 90 days in the validation cohort.

In the original cohort, the incidence of the primary outcome, which in this study refers to in-hospital mortality at both 30 and 90 days, was carefully analyzed across different subgroups of patients categorized based on their MCV levels. This analysis was performed using Kaplan-Meier survival curves, a widely utilized method for estimating the probability of survival over time and for comparing survival between different groups. The results of this analysis, which are clearly depicted in Fig 2A and 2B, revealed that patients with the highest MCV levels had a significantly higher risk of mortality at both 30 and 90 days.

Statistical testing confirmed the robustness of this finding, with a p-value less than 0.0001, indicating a highly significant association between elevated MCV levels and increased mortality risk. These findings suggest that MCV may serve as an important clinical marker for identifying patients at higher risk of adverse outcomes in the ICU setting, particularly in critically ill individuals with CKD.

A similar trend in the relationship between MCV levels and mortality risk was observed in the validation cohort, which was derived from the eICU-CRD. As shown in Fig 2C and 2D, the Kaplan-Meier survival curves for the validation cohort also demonstrated that higher MCV levels were associated with a substantially increased risk of mortality at both 30 and 90 days.

This parallel result across two independent cohorts strengthens the validity and generalizability of the findings, suggesting that the observed association between MCV and mortality is consistent across different patient populations and healthcare settings. These findings further support the potential role of MCV as a valuable prognostic marker for predicting patient outcomes in critically ill patients with CKD.

#### 3.2.2. Cox proportional Hazard ratios for all-cause mortality.

Cox proportional hazards analysis was conducted to assess the relationship between MCV and in-hospital mortality (**Table 2**). In the original cohort, when MCV was treated as a continuous variable, multiple models identified it as a significant risk factor for 30-day mortality. In the unadjusted model, the HR was 1.03 (95% CI: 1.02–1.04, p < 0.0001), and in the fully adjusted model, the HR was 1.04 (95% CI: 1.02–1.05, p < 0.0001). Similarly, multiple models have identified MCV as a significant risk factor for 90-day mortality.

**Table 2:** Cox regression analysis of MCV and mortality in CKD patients in the original cohort.

Categories	crude model		Model 1		Model 2		Model 3	
	95%CI	P	95%CI	P	95%CI	P	95%CI	P
Hospital mortality in the 30 days								
Continuous variable per unit	1.03(1.03,1.04)	<0.0001	1.03(1.02,1.04)	<0.0001	1.02(1.01,1.02)	<0.0001	1.04(1.03,1.05)	<0.0001
Quartile								
Q1	ref		ref		ref		ref	
Q2	1.00 (0.86,1.15)	0.96	0.95(0.83,1.10)	0.53	0.84(0.73,0.97)	0.02	0.95(0.81,1.11)	0.52
Q3	1.28(1.11,1.46)	<0.001	1.21(1.05,1.38)	0.01	1.06(0.93,1.22)	0.39	1.24(1.06,1.46)	0.01
Q4	1.83(1.62,2.08)	<0.0001	1.73(1.53,1.97)	<0.0001	1.31(1.15,1.49)	<0.0001	1.61(1.35,1.94)	<0.0001
p for trend		<0.0001		<0.0001		<0.0001		<0.0001
Hospital mortality in the 90 days								
Continuous variable per unit	1.03(1.03,1.04)	<0.0001	1.03(1.02,1.04)	<0.0001	1.02(1.01,1.02)	<0.0001	1.04(1.03,1.05)	<0.0001
Quartile								
Q1	ref		ref		ref		ref	
Q2	1(0.87,1.15)	0.98	0.96(0.83,1.10)	0.55	0.84(0.73,0.97)	0.02	0.94(0.81,1.10)	0.47
Q3	1.29(1.13,1.47)	<0.001	1.22(1.07,1.39)	0.003	1.08(0.95,1.23)	0.24	1.25(1.07,1.47)	0.005
Q4	1.81(1.60,2.05)	<0.0001	1.71(1.51,1.94)	<0.0001	1.31(1.15,1.48)	<0.0001	1.61(1.35,1.92)	<0.0001
p for trend		<0.0001		<0.0001		<0.0001		<0.001

crude model: unadjusted.

model 1 adjusted for sex, age, weight.

model 2: adjusted for sex, age, weight, CCI, OASIS, SAPS II, SOFA.

model 3: adjusted for sex, age, weight, CCI, OASIS, SAPS II, SOFA, Sodium, FBG, Serum creatinine, WBC, RBC, Platelet, Hemoglobin, Sepsis, diabetes, Arterial fibrillation, Respiratory failure, Heart failure, Epinephrine, Dopamine, Vasopressin.

When MCV was considered as a categorical variable in the original cohort, the risk of 30-day in-hospital mortality was significantly higher in the highest MCV group than in the lowest MCV group. In the unadjusted model, the HR was 1.72 (95% CI: 1.42–2.03, p < 0.0001). In the fully adjusted model, the HR was 1.71 (95% CI: 1.36–2.16, p < 0.0001). Similarly, multiple models indicated that patients in the highest MCV group had a significantly higher risk of in-hospital death within 90 days than those in the lower MCV group.

Similar results were observed in the multivariate Cox proportional hazards analysis of the validation cohort, which also evaluated the association between MCV and mortality (S2 Table).

#### 3.2.3. Analysis of nonlinear relationships.

In this study, an RCS analysis was applied to investigate the potential nonlinear relationship between MCV levels and in-hospital mortality, which is the primary outcome of interest. The use of RCS is particularly valuable in this context, as it allows for the modeling of complex, non-linear associations that might exist between variables, while still maintaining flexibility in the estimation process. By using RCS, we aimed to better understand how MCV levels might influence patient outcomes over time, taking into account the possibility of both linear and nonlinear effects.

**Fig 3:**
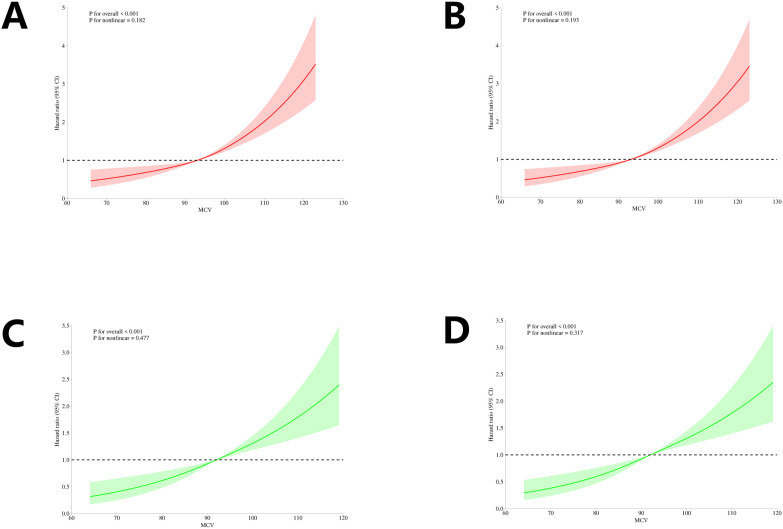
RCS for all-cause mortality. (A) 30-day mortality in the original cohort; (B) 90-day mortality in the original cohort; (C) 30-day mortality in the validation cohort; and (D) 90-day mortality in the validation cohort.

In the original cohort, as shown in Fig 3A and 3B, the analysis revealed that MCV levels exhibited a linear relationship with both 30-day and 90-day in-hospital mortality in patients with CKD. This means that as MCV levels increased, the risk of mortality also increased in a relatively consistent manner across the full range of MCV values. The P_nonlinear values for both 30-day and 90-day mortality were found to be 0.182 and 0.193, respectively, both of which were greater than the conventional threshold of 0.05, suggesting that the relationship between MCV and mortality did not exhibit significant nonlinear characteristics in this cohort.

Similarly, in the validation cohort, as presented in Fig 3C and 3D, MCV showed a linear association with both 30-day and 90-day in-hospital mortality. The P_nonlinear values for the validation cohort were 0.477 and 0.317, respectively, both of which were also above the threshold of 0.05. These values further support the notion that there was no significant nonlinear relationship between MCV and mortality in this cohort, reinforcing the findings from the original cohort.

Taken together, the results from both the original and validation cohorts suggest that MCV has a linear association with in-hospital mortality in patients with CKD, and that this relationship does not appear to be influenced by complex nonlinear patterns.

### 3.3. Subgroup analysis

Table 3 presents a detailed stratified analysis that examines the relationship between MCV levels and mortality risk in the original cohort, with a particular focus on highlighting the predictive value of MCV for in-hospital mortality outcomes across various subgroups of patients. The stratification in this analysis allows for a more nuanced understanding of how MCV might serve as a prognostic marker in different patient populations, including those with varying degrees of CKD severity, comorbid conditions, or other clinical characteristics that might influence patient outcomes.

**Table 3:** HRs for all-cause mortality in different subgroups in the original cohort.

Character	HR (95% CI)	p	p for interaction
Age			0.004
> 65	1.034(1.026,1.041)	<0.0001	
≤ 65	1.058(1.043,1.073)	<0.0001	
Sex			0.166
Female	1.034(1.023,1.045)	<0.0001	
Male	1.044(1.035,1.052)	<0.0001	
Heart failure			0.006
No	1.052(1.041,1.063)	<0.0001	
Yes	1.033(1.025,1.041)	<0.0001	
Respiratory failure			0.919
No	1.033(1.023,1.044)	<0.0001	
Yes	1.034(1.025,1.043)	<0.0001	
Arterial fibrillation			< 0.001
No	1.051(1.041,1.061)	<0.0001	
Yes	1.027(1.018,1.036)	<0.0001	
Sepsis			0.913
No	1.033(1.023,1.042)	<0.0001	
Yes	1.032(1.022,1.042)	<0.0001	
Stroke			0.612
No	1.040(1.034,1.047)	<0.0001	
Yes	1.033(1.004,1.063)	0.028	
Diabetes			0.456
No	1.042(1.032,1.052)	<0.0001	
Yes	1.037(1.028,1.046)	<0.0001	
Paraplegia			0.591
No	1.040(1.033,1.047)	<0.0001	
Yes	1.018(0.936,1.101)	0.667	

The findings from this stratified analysis consistently demonstrated that higher MCV levels were associated with an increased risk of death among patients with CKD, suggesting that MCV could potentially be used as a reliable biomarker to predict poor outcomes in this vulnerable patient group.

In addition to the results observed in the original cohort, a similar trend in the relationship between MCV and mortality was also observed in the validation cohort. This is shown in S3 Table, which presents the corresponding stratified analysis for the validation cohort extracted from the eICU-CRD. The consistency of the findings across both the original and validation cohorts further strengthens the reliability and generalizability of the observed association, supporting the idea that MCV is a robust predictor of in-hospital mortality in patients with CKD.

This replication of results across two independent datasets underscores the potential clinical utility of MCV as a prognostic tool for identifying patients at higher risk of adverse outcomes in the ICU.

### 3.4. Sensitivity analysis

To ensure the reliability and stability of the research findings and verify the robustness of our results, a comprehensive approach involving multiple sensitivity analyses was meticulously adopted. Specifically, one of the key modifications involved altering the stratification method of MCV. Initially, the MCV was stratified based on the Interquartile Range, but to further explore its potential influence, the stratification was changed to tertile. Subsequently, advanced statistical methods were employed, with Cox Proportional Hazard Ratios being utilized to in-depth analyze the intricate relationship between different stratifications of MCV and the prognosis of CKD patients. Additionally, Kaplan-Meier Survival Analysis was also conducted on MCV stratified by tertile to provide a more comprehensive assessment of its association with patient prognosis.

The results of sensitivity analysis suggest that, in the original cohort, the Cox Proportional Hazard Ratios (S4 Table) and Kaplan-Meier Survival Analysis (S1 Fig) both demonstrated statistically significant and clinically meaningful results. They revealed that higher tertile of MCV were associated with poor 30-day and 90-day prognosis in CKD patients (p < 0.001). This indicated that MCV, when stratified by tertile, could serve as a potential prognostic indicator for short-term outcomes in CKD patients within the original cohort. The statistical significance (p < 0.001) further emphasized the strength and reliability of this association, suggesting that it was unlikely to have occurred by chance and highlighting the importance of considering MCV levels in the clinical evaluation of CKD patients’ prognosis.

Similarly, in the validation cohort, the Cox Proportional Hazard Ratios (S5 Table) and Kaplan-Meier Survival Analysis (S1 Fig) also showed consistent results. They indicated that higher tertile of MCV were associated with poor 30-day and 90-day prognosis in CKD patients (p < 0.001). This consistency between the original and validation cohorts strengthens the validity and generalizability of the findings. It suggests that the observed association between higher tertile MCV and poor prognosis in CKD patients is not cohort-specific but rather a more universal phenomenon. The highly significant p-value (p < 0.001) once again underscores the robustness of this relationship, providing further evidence of the potential clinical utility of MCV as a prognostic marker in CKD patients.

## 4. Discussion

The current study aimed to explore the association between MCV levels and mortality in patients with CKD admitted to the ICU by leveraging a multicenter approach utilizing data from the eICU-CRD and MIMIC-IV databases. Our findings revealed that higher MCV levels were significantly associated with increased 30-day and 90-day in-hospital mortality in patients with CKD in the ICU. This association persisted after adjusting for various confounding factors, including demographic information, comorbidities, laboratory parameters, illness severity scores, and medication use.

Kaplan–Meier survival analysis demonstrated that patients with higher MCV levels had a significantly increased risk of mortality at both 30 and 90 days. Cox proportional hazards models further confirmed that MCV, whether treated as a continuous or categorical variable, was a significant risk factor for in-hospital mortality. Notably, the fully adjusted models showed that for each unit increase in MCV, there was a corresponding increase in the HR for mortality. Subgroup analyses also consistently indicated that higher MCV levels were associated with a higher risk of death across different patient groups.

The MCV represents the average volume of a single RBC and is a key parameter in the morphological classification of anemias [13]. It is typically measured in femtoliters (fL). Blood tests, often utilizing automated cell analyzers, are commonly used to determine the MCV. For instance, a new electrochemical method for detecting RBC has been developed that relies on single-particle collision events. This method converts quantitative information on the size and concentration of RBCs, obtained from the current decrease caused by RBC collisions on the electrode, into MCV for diagnostic purposes [14].

MCV plays a crucial role in the diagnosis of anemia in patients with CKD. Anemia can be classified into microcytic (MCV < 80 fL), normocytic (MCV 80–100 fL), and macrocytic (MCV > 100 fL) categories, which helps in determining the underlying cause of anemia. For instance, in a study of 4,129 patients newly diagnosed with anemia in primary care, the MCV-guided classification was initially thought to aid in defining anemia etiology. However, it was found that in 59 of 365 microcytic patients (16%), the anemia etiology did not align with the assumption that microcytic anemia excludes conditions such as vitamin B12 deficiency, folic acid deficiency, suspected hemolysis, and suspected bone marrow disease. Similarly, in 233 of 259 macrocytic patients (90%), anemia etiology contradicted the assumption that macrocytic anemia excludes iron deficiency anemia, chronic anemia, and renal anemia [15]. In screening for β-thalassemia carriers in the Guangdong population of China, it is strongly recommended to evaluate both MCV and mean corpuscular hemoglobin. Among the 6,779 β-thalassemia carriers, combining MCV and mean corpuscular hemoglobin reduced the false-negative screening rate to 0.22% [16].

Anemia is a common complication of CKD. Data from the National Health and Nutrition Examination Survey conducted between 2007 and 2010 indicated that an estimated 14.0% of the U.S. adult population had CKD, and anemia was twice as prevalent in individuals with CKD (15.4%) as in the general population (7.6%). The prevalence of anemia increases with CKD stage, ranging from 8.4% at stage 1 to 53.4% at stage 5 [17]. In a study of 2,198 non-dialysis patients with CKD in Korea, the overall prevalence of anemia was 45.0%. Independent risk factors for anemia include diabetic nephropathy, CKD stages, body mass index, smoking, leukocyte count, serum albumin, iron markers, and calcium and phosphorus concentrations [18]. A multicenter cross-sectional study of predialysis CKD patients in Saudi Arabia found elevated anemia rates, defined as hemoglobin levels < 12 g/dL, across different CKD stages. The prevalence of anemia was 42%, 33%, 48%, 71%, and 82% in stages 1–5, respectively [19].

The relationship between MCV and CKD has been the subject of ongoing research. In a retrospective study involving 1,075 patients with stage 3–5 CKD, the interaction between MCV and red cell distribution width (RDW) was examined. Patients were categorized into four groups based on RDW and MCV levels. The adjusted HR for all-cause mortality in the group with high RDW and low MCV compared to the group with low RDW and low MCV was 1.44 (95% confidence interval [CI], 1.14–2.12, p = 0.02), suggesting that the combination of high RDW and low MCV is associated with an increased risk of all-cause mortality [20]. Another study of 1,439 patients with stages 3–5 CKD found that the high-MCV group was linked to higher risks of all-cause mortality (HR, 2.19; 95% CI, 1.62–2.96; p < 0.001), cardiovascular mortality (HR, 3.57; 95% CI, 1.80–7.06; p < 0.001), and infection-related mortality (HR, 2.22; 95% CI, 1.41–3.49; p = 0.001) than the low-MCV group [8].

The mechanism underlying the association between MCV and mortality remains unclear. One potential explanation is that MCV may reflect underlying nutritional deficiencies or chronic diseases. Elevated MCV is often linked to macrocytic anemia, which can result from deficiencies in vitamin B12 or folate, which are essential for both DNA synthesis and RBC production. These deficiencies can impair erythropoiesis and increase RBC turnover, thereby contributing to a higher risk of mortality [21,22]. Additionally, high MCV levels are associated with alcohol consumption, which can cause liver damage and lead to macrocytosis. Liver dysfunction may impair the metabolism of folate and vitamin B12, further exacerbating macrocytic anemia and increasing mortality risk. This is particularly relevant in patients with chronic liver disease, where elevated MCV has been linked to higher all-cause and liver cancer mortalities [6].

In patients with CKD, elevated MCV has been associated with increased mortality, possibly due to the complex interaction between anemia, inflammation, and cardiovascular disease. CKD patients frequently develop anemia due to reduced erythropoietin production, and elevated MCV may reflect a more severe form of anemia or underlying inflammation, both of which are known to be linked to higher mortality rates [23,11].

Moreover, the interaction between MCV and RDW has been shown to affect mortality risk. Studies have indicated that the combination of high MCV and high RDW is significantly associated with an increased risk of mortality, suggesting that these parameters together may reflect more severe underlying pathophysiological processes, such as oxidative stress or inflammation, which contribute to adverse outcomes [20,12]. In the context of cardiovascular diseases, elevated MCV has been linked to poor outcomes following procedures such as peripheral angioplasty with stenting. This may be due to the relationship between MCV and platelet reactivity, as high MCV levels correlate with increased platelet reactivity, potentially leading to higher rates of restenosis or thrombotic events, which in turn increases mortality risk [24].

Overall, the association between MCV and mortality is likely multifactorial and involves factors such as nutritional deficiencies, chronic disease states, inflammation, and hematological abnormalities. Further research is needed to clarify the precise mechanisms and investigate whether interventions targeting these underlying factors could improve clinical outcomes in patients with elevated MCV [8,25].

To ensure the reliability and stability of the research findings and verify the robustness of our results, a comprehensive approach involving multiple sensitivity analyses was meticulously adopted. The results of sensitivity analysis suggest that, in both the original and validation cohorts, the Cox Proportional Hazard Ratios and Kaplan-Meier Survival Analysis both demonstrated statistically significant and clinically meaningful results, revealing that higher tertile of MCV were associated with poor 30-day and 90-day prognosis in CKD patients (p < 0.001), which strengthens the validity and generalizability of the findings and highlights the potential clinical utility of MCV as a prognostic marker in CKD patients.

This is the first multicenter study to examine the relationship between MCV and mortality in ICU patients with CKD. Compared with single-center studies, multicenter studies offer several advantages, including a larger sample size and a more diverse population, which can yield more robust and generalizable results. This is particularly important when investigating treatments for diseases that are rare or affect specific populations. Multicenter studies help reduce bias, as the findings are less likely to be influenced by the practices or preferences of a single research team or location. Consequently, the results tend to be more accurate and reliable.

The strengths of our study include its large sample size, multicenter design, and comprehensive adjustment for potential confounders. However, this study has several limitations. First, as a retrospective study, it is susceptible to inherent biases, such as selection bias and residual confounding. Second, we were unable to account for potential unmeasured confounders that may have influenced the association between MCV and mortality.

## 5. Conclusion

In summary, our multicenter study demonstrated that elevated MCV levels are significantly associated with higher 30-day and 90-day in-hospital mortality in patients with CKD admitted to the ICU. This study is the first to explore this relationship through a multicenter approach, providing more generalizable and robust findings than those of previous single-center studies. Although the exact mechanisms underlying this association remain to be elucidated, our results suggest that MCV could serve as a potential biomarker for risk stratification and prognosis assessment in patients with CKD within the ICU setting. Future research should focus on validating these findings and investigating the underlying pathophysiological processes.

## Supporting information

S1 Fig Kaplan-Meier curves of MCV and mortality at different tertiles. (A) Death within 30 days in the original cohort; (B) death within 90 days in the original cohort; (C) death within 30 days in the validation cohort; and (D) death within 90 days in the validation cohort.
(TIF)

S1 Table Baseline characteristics of survivors and deaths in validation cohort. Data are presented Standard Deviation (SE) or frequencies (percentages). Abbreviation: MCV, mean corpuscular volume; SOFA, sequential organ failure assessment; CCI, Charlson comorbidity index; SAPSII, simplified acute physiological score II; OASIS, oxford acute severity of illness score; WBC, white blood cell; RBC, red blood cell; FBG, fasting blood glucose.
(DOCX)

S2 Table Cox regression analysis of MCV and mortality in CKD patients in the validation cohort. crude model: unadjusted. model 1: adjusted for sex, age, weight. model 2: adjusted for sex, age, weight, CCI, OASIS, SAPS II, SOFA. model 3: adjusted for sex, age, weight, CCI, OASIS, SAPS II, SOFA, Sodium, FBG, Serum creatinine, WBC, RBC, Platelet, Hemoglobin, Sepsis, diabetes, Arterial fibrillation, Respiratory failure, Heart failure, Epinephrine, Dopamine, Vasopressin.
(DOCX)

S3 Table HRs for all-cause mortality in different subgroups in the validation cohort. (DOCX)

S4 Table Cox regression analysis of MCV tertiles and mortality in CKD patients in the original cohort. crude model: unadjusted. model 1: adjusted for sex, age, weight. model 2: adjusted for sex, age, weight, CCI, OASIS, SAPS II, SOFA. model 3: adjusted for sex, age, weight, CCI, OASIS, SAPS II, SOFA, Sodium, FBG, Serum creatinine, WBC, RBC, Platelet, Hemoglobin, Sepsis, diabetes, Arterial fibrillation, Respiratory failure, Heart failure, Epinephrine, Dopamine, Vasopressin.
(DOCX)

S5 Table Cox regression analysis of MCV tertiles and mortality in CKD patients in the validation cohort. crude model: unadjusted. model 1: adjusted for sex, age, weight. model 2: adjusted for sex, age, weight, CCI, OASIS, SAPS II, SOFA. model 3: adjusted for sex, age, weight, CCI, OASIS, SAPS II, SOFA, Sodium, FBG, Serum creatinine, WBC, RBC, Platelet, Hemoglobin, Sepsis, diabetes, Arterial fibrillation, Respiratory failure, Heart failure, Epinephrine, Dopamine, Vasopressin.
(DOCX)
