# Supplementary figures and images for: Association between mean corpuscular volume and mortality in chronic kidney disease ICU patients: A retrospective multicenter cohort study
Source: PLoS One. 2025 Aug 13;20(8):e0328980. doi: 10.1371/journal.pone.0328980 (PMC12349715; doi:10.1371/journal.pone.0328980)

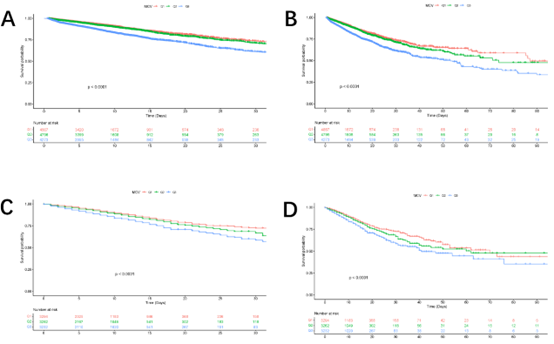

Supplement: S1 Fig — (A) Death within 30 days in the original cohort; (B) death within 90 days in the original cohort; (C) death within 30 days in the validation cohort; and (D) death within 90 days in the validation cohort. (TIF) [file pone.0328980.s001.tif]
