# Supplementary material for: Association between mean corpuscular volume and mortality in chronic kidney disease ICU patients: A retrospective multicenter cohort study
Source: PLoS One. 2025 Aug 13;20(8):e0328980. doi: 10.1371/journal.pone.0328980 (PMC12349715; doi:10.1371/journal.pone.0328980)
Supplement: S1 Table — Data are presented Standard Deviation (SE) or frequencies (percentages). Abbreviation: MCV, mean corpuscular volume; SOFA, sequential organ failure assessment; CCI, Charlson comorbidity index; SAPSII, simplified acute physiological score II; OASIS, oxford acute severity of illness score; WBC, white blood cell; RBC, red blood cell; FBG, fasting blood glucose. (DOCX) [file pone.0328980.s002.docx]

**Table S1**. Baseline characteristics of survivors and deaths in validation cohort.

| Variable | Total (n=9788) | Death (n=1221) | Alive (n=8567) | p.value |
| --- | --- | --- | --- | --- |
| MCV | 92.19 ± 7.58 | 93.53 ± 8.05 | 92.00 ± 7.49 | <0.0001 |
| Sex |  |  |  | 0.30 |
| Female | 4371(44.66) | 528(43.24) | 3843(44.86) |  |
| Male | 5417(55.34) | 693(56.76) | 4724(55.14) |  |
| Age | 66.87 ± 14.85 | 70.47 ± 13.53 | 66.35 ± 14.96 | <0.0001 |
| Weight | 84.92 ± 27.68 | 83.90 ± 28.42 | 85.07 ± 27.57 | 0.18 |
| Comorbidities |  |  |  |  |
| Heart failure |  |  |  | <0.01 |
| No | 7527(76.90) | 898(73.55) | 6629(77.38) |  |
| Yes | 2261(23.10) | 323(26.45) | 1938(22.62) |  |
| Respiratory failure |  |  |  | <0.0001 |
| No | 6920(70.70) | 587(48.08) | 6333(73.92) |  |
| Yes | 2868(29.30) | 634(51.92) | 2234(26.08) |  |
| Arterial fibrillation |  |  |  | <0.0001 |
| No | 8186(83.63) | 919(75.27) | 7267(84.83) |  |
| Yes | 1602(16.37) | 302(24.73) | 1300(15.17) |  |
| Diabetes |  |  |  | 0.99 |
| No | 6772(69.19) | 844(69.12) | 5928(69.20) |  |
| Yes | 3016(30.81) | 377(30.88) | 2639(30.80) |  |
| Paraplegia |  |  |  | 1.00 |
| No | 9777(99.89) | 1220(99.92) | 8557(99.88) |  |
| Yes | 11(0.11) | 1(0.08) | 10(0.12) |  |
| Sepsis |  |  |  | <0.0001 |
| No | 7729(78.96) | 804(65.85) | 6925(80.83) |  |
| Yes | 2059(21.04) | 417(34.15) | 1642(19.17) |  |
| Stroke |  |  |  | <0.0001 |
| No | 9258(94.59) | 1124(92.06) | 8134(94.95) |  |
| Yes | 530(5.41) | 97(7.94) | 433(5.05) |  |
| SAPSII | 35.00(27.00,45.00) | 48.00(38.00,61.00) | 34.00(27.00,42.00) | <0.0001 |
| SOFA | 6.00(5.00,8.00) | 9.00(7.00,12.00) | 6.00(5.00,8.00) | <0.0001 |
| CCI | 6.00(4.00,8.00) | 6.00(5.00,8.00) | 6.00(4.00,8.00) | <0.0001 |
| OASIS | 26.00(20.00,33.00) | 34.00(27.00,42.00) | 25.00(20.00,31.00) | <0.0001 |
| Laboratory tests |  |  |  |  |
| WBC, K/uL | 11.10(8.00,15.60) | 14.00(9.50,20.20) | 10.80(7.82,15.00) | <0.0001 |
| RBC, m/uL | 3.60 ± 0.73 | 3.58 ± 0.72 | 3.60 ± 0.73 | 0.22 |
| Hemoglobin, g/dL | 10.76 ± 2.04 | 10.72 ± 2.05 | 10.77 ± 2.03 | 0.47 |
| Platelet, K/uL | 194.00(145.00,255.00) | 191.00(132.00,262.00) | 195.00(146.50,254.00) | 0.29 |
| FBG, mg/dL | 170.00(129.00,238.00) | 181.00(135.00,253.00) | 169.00(128.00,235.00) | 0.06 |
| Sodium, mEq/L | 138.66 ± 5.04 | 139.24 ± 6.03 | 138.58 ± 4.87 | <0.001 |
| SCr, mg/dL | 3.05(1.67,5.80) | 3.23(2.03,5.14) | 3.00(1.61,5.94) | <0.001 |
| Drug use |  |  |  |  |
| Vasopressin |  |  |  | <0.0001 |
| No | 9282(94.83) | 1006(82.39) | 8276(96.60) |  |
| Yes | 506(5.17) | 215(17.61) | 291(3.40) |  |
| Dopamine |  |  |  | <0.0001 |
| No | 9227(94.27) | 1076(88.12) | 8151(95.14) |  |
| Yes | 561(5.73) | 145(11.88) | 416(4.86) |  |
| Epinephrine |  |  |  | <0.0001 |
| No | 9256(94.56) | 1082(88.62) | 8174(95.41) |  |
| Yes | 532(5.44) | 139(11.38) | 393(4.59) |  |

Data are presented Standard Deviation (SE) or frequencies (percentages).

Abbreviation: MCV, mean corpuscular volume; SOFA, sequential organ failure assessment; CCI, Charlson comorbidity index; SAPSII, simplified acute physiological score II; OASIS, oxford acute severity of illness score; WBC, white blood cell; RBC, red blood cell; FBG, fasting blood glucose; SCr, Serum Creatinine
