# Supplementary material for: Association between mean corpuscular volume and mortality in chronic kidney disease ICU patients: A retrospective multicenter cohort study
Source: PLoS One. 2025 Aug 13;20(8):e0328980. doi: 10.1371/journal.pone.0328980 (PMC12349715; doi:10.1371/journal.pone.0328980)
Supplement: S2 Table — crude model: unadjusted. model 1: adjusted for sex, age, weight. model 2: adjusted for sex, age, weight, CCI, OASIS, SAPS II, SOFA. model 3: adjusted for sex, age, weight, CCI, OASIS, SAPS II, SOFA, Sodium, FBG, Serum creatinine, WBC, RBC, Platelet, Hemoglobin, Sepsis, diabetes, Arterial fibrillation, Respiratory failure, Heart failure, Epinephrine, Dopamine, Vasopressin. (DOCX) [file pone.0328980.s003.docx]

Table S2. Cox regression analysis of MCV and mortality in CKD patients in the validation cohort.

| Categories | crude model | | Model 1 | | Model 2 | | Model 3 | |
| --- | --- | --- | --- | --- | --- | --- | --- | --- |
|  | 95%CI | P | 95%CI | P | 95%CI | P | 95%CI | P |
| Hospital mortality in the 30 days |  |  |  |  |  |  |  |  |
| Continuous variable per unit | 1.03(1.02,1.04) | <0.0001 | 1.03(1.02,1.03) | <0.0001 | 1.01(1.00,1.02) | 0.001 | 1.04(1.02,1.05) | <0.0001 |
| Quartile |  |  |  |  |  |  |  |  |
| Q1 | ref |  | ref |  | ref |  | ref |  |
| Q2 | 1.08(0.91,1.29) | 0.39 | 1.05(0.88,1.25) | 0.57 | 1(0.83,1.19) | 0.96 | 1.16(0.95,1.40) | 0.14 |
| Q3 | 1.33(1.12,1.57) | <0.001 | 1.24(1.05,1.47) | 0.01 | 1.18(1.00,1.40) | 0.05 | 1.42(1.15,1.74) | <0.001 |
| Q4 | 1.72(1.47,2.03) | <0.0001 | 1.62(1.38,1.90) | <0.0001 | 1.26(1.07,1.49) | 0.01 | 1.71(1.36,2.16) | <0.0001 |
| p for trend |  | <0.0001 |  | <0.0001 |  | <0.0001 |  | <0.0001 |
| Hospital mortality in the 90 days |  |  |  |  |  |  |  |  |
| Continuous variable per unit | 1.03(1.02,1.04) | <0.0001 | 1.03(1.02,1.03) | <0.0001 | 1.01(1.00,1.02) | 0.002 | 1.04(1.02,1.05) | <0.0001 |
| Quartile |  |  |  |  |  |  |  |  |
| Q1 | ref |  | ref |  | ref |  | ref |  |
| Q2 | 1.08(0.91,1.28) | 0.37 | 1.06(0.89,1.25) | 0.54 | 0.99(0.84,1.18) | 0.92 | 1.18(0.97,1.42) | 0.09 |
| Q3 | 1.33(1.13,1.56) | <0.001 | 1.24(1.05,1.46) | 0.01 | 1.18(1.00,1.39) | 0.05 | 1.45(1.19,1.77) | <0.001 |
| Q4 | 1.71(1.46,2.00) | <0.0001 | 1.61(1.37,1.88) | <0.0001 | 1.25(1.06,1.46) | 0.01 | 1.73(1.38,2.16) | <0.0001 |
| p for trend |  | <0.0001 |  | <0.0001 |  | <0.0001 |  | <0.001 |

crudel model: unadjusted

model 1 adjusted for sex, age, weight

model 2: adjusted for sex, age, weight, CCI, OASIS, SAPS II, SOFA

model 3: adjusted for sex, age, weight, CCI, OASIS, SAPS II, SOFA, Sodium, FBG, Serum creatinine, WBC, RBC, Platelet, Hemoglobin, Sepsis, diabetes, Arterial fibrillation, Respiratory failure, Heart failure, Epinephrine, Dopamine, Vasopressin
