# Supplementary material for: Association between mean corpuscular volume and mortality in chronic kidney disease ICU patients: A retrospective multicenter cohort study
Source: PLoS One. 2025 Aug 13;20(8):e0328980. doi: 10.1371/journal.pone.0328980 (PMC12349715; doi:10.1371/journal.pone.0328980)
Supplement: S3 Table — (DOCX) [file pone.0328980.s004.docx]

**Table S3.** HRs for all-cause mortality in different subgroups in the validation cohort.

| Character | HR (95% CI) | p | p for interaction |
| --- | --- | --- | --- |
| Age |  |  | 0.347 |
| >65 | 1.028(1.017,1.038) | <0.0001 |  |
| ≤65 | 1.020(1.006,1.033) | 0.004 |  |
| Sex |  |  | 0.33 |
| Female | 1.023(1.011,1.035) | <0.001 |  |
| Male | 1.031(1.020,1.042) | <0.0001 |  |
| Heart failure |  |  | 0.424 |
| No | 1.022(1.006,1.038) | 0.008 |  |
| Yes | 1.029(1.020,1.039) | <0.0001 |  |
| Respiratory failure |  |  | 0.088 |
| No | 1.032(1.020,1.044) | <0.0001 |  |
| Yes | 1.017(1.006,1.029) | 0.002 |  |
| Arterial fibrillation |  |  | 0.778 |
| No | 1.024(1.008,1.041) | 0.004 |  |
| Yes | 1.027(1.018,1.036) | <0.0001 |  |
| Sepsis |  |  | 0.01 |
| No | 1.032(1.022,1.043) | <0.0001 |  |
| Yes | 1.010(0.997,1.024) | 0.142 |  |
| Stroke |  |  | 0.344 |
| No | 1.029(1.020,1.037) | <0.0001 |  |
| Yes | 1.013(0.983,1.045) | 0.398 |  |
| Diabetes |  |  | 0.209 |
| No | 1.031(1.021,1.040) | <0.0001 |  |
| Yes | 1.019(1.004,1.034) | 0.011 |  |
