# Supplementary material for: Association between mean corpuscular volume and mortality in chronic kidney disease ICU patients: A retrospective multicenter cohort study
Source: PLoS One. 2025 Aug 13;20(8):e0328980. doi: 10.1371/journal.pone.0328980 (PMC12349715; doi:10.1371/journal.pone.0328980)
Supplement: S4 Table — crude model: unadjusted. model 1: adjusted for sex, age, weight. model 2: adjusted for sex, age, weight, CCI, OASIS, SAPS II, SOFA. model 3: adjusted for sex, age, weight, CCI, OASIS, SAPS II, SOFA, Sodium, FBG, Serum creatinine, WBC, RBC, Platelet, Hemoglobin, Sepsis, diabetes, Arterial fibrillation, Respiratory failure, Heart failure, Epinephrine, Dopamine, Vasopressin. (DOCX) [file pone.0328980.s005.docx]

Table S4. Cox regression analysis of MCV tertiles and mortality in CKD patients in the original cohort.

| Categories | crude model | | Model 1 | | Model 2 | | Model 3 | |
| --- | --- | --- | --- | --- | --- | --- | --- | --- |
|  | 95%CI | P | 95%CI | P | 95%CI | P | 95%CI | P |
| Hospital mortality in the 30 days |  |  |  |  |  |  |  |  |
| Quartile |  |  |  |  |  |  |  |  |
| Q1 | ref |  | ref |  | ref |  | ref |  |
| Q2 | 1.11(0.98,1.25) | 0.11 | 1.06(0.93,1.20) | 0.39 | 0.94(0.83,1.07) | 0.36 | 1.09(0.95,1.26) | 0.23 |
| Q3 | 1.79(1.60,2.01) | <0.0001 | 1.7(1.51,1.90) | <0.0001 | 1.33(1.18,1.49) | <0.0001 | 1.73(1.47,2.03) | <0.0001 |
| p for trend |  | <0.0001 |  | <0.0001 |  | <0.0001 |  | <0.0001 |
| Hospital mortality in the 90 days |  |  |  |  |  |  |  |  |
| Quartile |  |  |  |  |  |  |  |  |
| Q1 | ref |  | ref |  | ref |  | ref |  |
| Q2 | 1.12(0.99,1.26) | 0.08 | 1.06(0.94,1.20) | 0.34 | 0.95(0.84,1.07) | 0.40 | 1.09(0.95,1.26) | 0.20 |
| Q3 | 1.78(1.59,1.99) | <0.0001 | 1.69(1.51,1.89) | <0.0001 | 1.34(1.19,1.50) | <0.0001 | 1.74(1.49,2.03) | <0.0001 |
| p for trend |  | <0.0001 |  | <0.0001 |  | <0.0001 |  | <0.001 |

crude model: unadjusted

model 1 adjusted for sex, age, weight

model 2: adjusted for sex, age, weight, CCI, OASIS, SAPS II, SOFA

model 3: adjusted for sex, age, weight, CCI, OASIS, SAPS II, SOFA, Sodium, FBG, Serum creatinine, WBC, RBC, Platelet, Hemoglobin, Sepsis, diabetes, Arterial fibrillation, Respiratory failure, Heart failure, Epinephrine, Dopamine, Vasopressin
