# Supplementary material for: Association between mean corpuscular volume and mortality in chronic kidney disease ICU patients: A retrospective multicenter cohort study
Source: PLoS One. 2025 Aug 13;20(8):e0328980. doi: 10.1371/journal.pone.0328980 (PMC12349715; doi:10.1371/journal.pone.0328980)
Supplement: S5 Table — crude model: unadjusted. model 1: adjusted for sex, age, weight. model 2: adjusted for sex, age, weight, CCI, OASIS, SAPS II, SOFA. model 3: adjusted for sex, age, weight, CCI, OASIS, SAPS II, SOFA, Sodium, FBG, Serum creatinine, WBC, RBC, Platelet, Hemoglobin, Sepsis, diabetes, Arterial fibrillation, Respiratory failure, Heart failure, Epinephrine, Dopamine, Vasopressin. (DOCX) [file pone.0328980.s006.docx]

Table S5. Cox regression analysis of MCV tertiles and mortality in CKD patients in the validation cohort.

| Categories | crude model | | Model 1 | | Model 2 | | Model 3 | |
| --- | --- | --- | --- | --- | --- | --- | --- | --- |
|  | 95%CI | P | 95%CI | P | 95%CI | P | 95%CI | P |
| Hospital mortality in the 30 days |  |  |  |  |  |  |  |  |
| Quartile |  |  |  |  |  |  |  |  |
| Q1 | ref |  | ref |  | ref |  | ref |  |
| Q2 | 1.17(1.01,1.36) | 0.04 | 1.13(0.97,1.31) | 0.12 | 1.09(0.94,1.27) | 0.26 | 1.22(1.03,1.44) | 0.02 |
| Q3 | 1.62(1.41,1.87) | <0.0001 | 1.52(1.32,1.75) | <0.0001 | 1.27(1.10,1.47) | <0.001 | 1.62(1.34,1.96) | <0.0001 |
| p for trend |  | <0.0001 |  | <0.0001 |  | <0.0001 |  | <0.0001 |
| Hospital mortality in the 90 days |  |  |  |  |  |  |  |  |
| Quartile |  |  |  |  |  |  |  |  |
| Q1 | ref |  | ref |  | ref |  | ref |  |
| Q2 | 1.17(1.01,1.36) | 0.03 | 1.13(0.97,1.30) | 0.11 | 1.09(0.94,1.26) | 0.27 | 1.22(1.04,1.44) | 0.02 |
| Q3 | 1.6(1.40,1.84) | <0.0001 | 1.5(1.31,1.73) | <0.0001 | 1.25(1.09,1.43) | 0.002 | 1.6(1.33,1.92) | <0.0001 |
| p for trend |  | <0.0001 |  | <0.0001 |  | <0.0001 |  | <0.001 |

crude model: unadjusted

model 1 adjusted for sex, age, weight

model 2: adjusted for sex, age, weight, CCI, OASIS, SAPS II, SOFA

model 3: adjusted for sex, age, weight, CCI, OASIS, SAPS II, SOFA, Sodium, FBG, Serum creatinine, WBC, RBC, Platelet, Hemoglobin, Sepsis, diabetes, Arterial fibrillation, Respiratory failure, Heart failure, Epinephrine, Dopamine, Vasopressin
